# Function and Dysfunction of Microglia during Brain Development: Consequences for Synapses and Neural Circuits

**DOI:** 10.3389/fnsyn.2017.00009

**Published:** 2017-05-10

**Authors:** Rosa C. Paolicelli, Maria T. Ferretti

**Affiliations:** ^1^IREM, Institute for Regenerative Medicine, University of ZurichZürich, Switzerland; ^2^ZNZ Neuroscience Center ZurichZürich, Switzerland

**Keywords:** microglia, synapses, brain development, synaptic function, synaptic pruning, neurodevelopmental disorders, infections, stress

## Abstract

Many diverse factors, ranging from stress to infections, can perturb brain homeostasis and alter the physiological activity of microglia, the immune cells of the central nervous system. Microglia play critical roles in the process of synaptic maturation and brain wiring during development. Any perturbation affecting microglial physiological function during critical developmental periods could result in defective maturation of synaptic circuits. In this review, we critically appraise the recent literature on the alterations of microglial activity induced by environmental and genetic factors occurring at pre- and early post-natal stages. Furthermore, we discuss the long-lasting consequences of early-life microglial perturbation on synaptic function and on vulnerability to neurodevelopmental and psychiatric disorders.

## Introduction: Microglia in the Developing Brain

Microglia are commonly defined as the immune cells of the central nervous system (CNS). Along with their well-established role as immediate responders to injuries and infections, microglia also play important functions in the healthy brain, critically interacting with other cell types to provide support and to monitor neuronal circuits. A growing body of evidence indicates a central role of microglia in successful brain development and wiring, thus setting the stage for a new exciting subfield of research (extensively reviewed in Bessis et al., 2007; Ji et al., 2013b; Kettenmann et al., 2013; Sierra et al., 2014; Casano and Peri, 2015; Wu et al., 2015). Microglia critically contribute to many physiological processes, such as developmentally regulated neuronal apoptosis, neurogenesis (Wakselman et al., 2008; Cunningham et al., 2013; Mazaheri et al., 2014; Shigemoto-Mogami et al., 2014) and synaptic pruning in physiological and pathological conditions (Blinzinger and Kreutzberg, 1968; Paolicelli et al., 2011; Schafer et al., 2012; Kim et al., 2016). Furthermore, microglia assist with brain wiring through the promotion of synaptic formation and maturation (Hoshiko et al., 2012; Ueno et al., 2013; Miyamoto et al., 2016). Due to their roles in synaptic development and function, microglia are currently regarded as the fourth component of the ‘quad-partite synapse,’ in addition to astrocytes and pre- and post-synaptic terminals (Paolicelli and Gross, 2011; Schafer et al., 2013). Microglia are extremely dynamic and responsive cells, and promptly respond to any homeostatic change (Davalos et al., 2005; Nimmerjahn et al., 2005). Pathological stimuli or brain insult profoundly affect microglial activity and switch their function from housekeepers to adaptive responders. The ensuing response perturbs microglial motility, morphology and function, thereby transiently compromising the execution of crucial physiological tasks. In the specific context of early brain maturation, it is becoming clear that severe perturbations of microglial activity, specifically during critical periods, might drastically delay the correct neuronal development and wiring of the brain. Here, we provide a summary of the recent literature supporting the importance of microglia for proper brain development. We focus on both environmental and genetic factors known to alter baseline microglial behavior, and discuss the consequent synaptic dysfunction and vulnerability to mental diseases.

## Understanding Microglial Function by Depletion Approaches

### Microglia Depletion Models

Removal of microglia during development has revealed a long-lasting impact on synaptic maturation and brain circuit formation. Both pharmacological and genetic methods have been adopted to deplete microglia from the brain. Common examples of pharmacological approaches to deplete microglia include the administration of toxic clodronate-containing liposomes (Buiting and Van Rooijen, 1994), and the inhibition of the Csf1 signaling pathway, crucial for microglial survival (Elmore et al., 2014; Squarzoni et al., 2014). Genetic depletion is achieved either by removing factors indispensable for microglial maturation and survival, such as PU.1 or CSF1R, or through the expression of ‘suicidal genes,’ such as diphtheria toxin receptor (DTR) or viral thymidine kinase (HSVTK) under the control of specific microglial promoters, as Cd11b or Cx3cr1 (see **Table 1**).

**Table 1:** Microglial depletion models.

	Targeted signaling	Model	Timing	Reported effects	Publications
Pharmacological depletion	Csf1R	Anti-CSF1R Antibody: treatment *in utero*	E6.5-E7.5	Defective development of TH-positive dopaminergic axons in the forebrain and altered positioning of Lhx6-expressing interneurons in the cortical plate	Squarzoni et al., 2014; Hoeffel et al., 2015
	Csf1R	CSF1R kinase inhibitor PLX3397: oral administration	2 months 12 months 18 months	No changes in motor or anxiety behaviors. Reduced escape latencies in the Barnes maze training. No changes in total brain volume.	Elmore et al., 2014
	Cell survival	Clodronate liposomes: dichloromethylene-bisphosphonate (Cl2MBP, Clodronate) induces apoptosis upon phagocytosis. CNS injection	PND2–4 Different time points	Long-lasting impairments in anxiety, social and locomotor behavior in rat	Nelson and Lenz, 2017; Buiting and Van Rooijen, 1994
Genetic depletion	Csf1R KO	Constitutive KO	Early embryonic depletion	Increased neuronal density in the cortex, elevated numbers of astrocytes but reduced numbers of oligodendrocytes	Dai et al., 2002; Ginhoux et al., 2010; Erblich et al., 2011
	PU.1 KO	Constitutive KO	Early embryonic depletion	Defasciculation of dorsal callosal axons. Exuberant extension of TH-positive axons into the subpallium	Iwasaki et al., 2005; Kierdorf et al., 2013; Pont-Lezica et al., 2014; Squarzoni et al., 2014
	Cx3cr1cre^ER^; iDTR	Inducible conditional depletion upon diphtheria toxin administration	PND19, PND30, PND60 PND12–14	PND19 and PND30: decreased spine formation and elimination. PND60: decreased learning-dependent spine formation, but not elimination PND12–14: increase in pro-inflammatory cytokines and chemokines in the cortex	Parkhurst et al., 2013; Bruttger et al., 2015
	Cd11b-HSVtk	Conditional depletion upon ganciclovir treatment. Thymidine kinase of herpes simplex virus (encoded by HSVtk) driven by the Cd11b	Adulthood	BBB damages	Heppner et al., 2005
	CNS-TGFβ KO	TGFβ depletion in the CNS	Early embryonic depletion	Motor abnormalities around PND100–120	Butovsky et al., 2014

*Table summarizing the major pharmacological and genetic models of microglia depletion. Csf1r, colony stimulating factor 1 receptor; PU.1, PU-box binding transcription factor 1; Cx3cr1, fractalkine receptor; iDTR, inducible diphtheria toxin; HSVtk, herpes simplex virus thymidine kinase; TGFβ, transforming growth factor-β.*

The advantage of pharmacological depletion methods is the possibility of treating any mouse strain, with a tight control on the timing of the treatment. However, clodronate liposomes do not cross the brain–blood barrier (BBB), thus requiring stereotaxic injections in the ventricles or in a selected brain structure. Because of the invasiveness of their administration, liposomes usage is not the method of choice for microglial depletion. Inhibitors of the CSF1R pathway, on the other hand, are easier to administer (via chow containing the inhibitor compound), but have significant side effects on other cells of the myeloid lineage, such as hematopoietic stems cells, macrophages, osteoclast, and mast cells (Thompson et al., 2015). In both cases, the pharmacological depletion is transient, since microglia are capable of rapidly repopulating the brain parenchyma within a few days of treatment (Elmore et al., 2014).

Genetic models such as constitutive knockout of critical transcription or survival factors (PU.1 KO, CSF1R KO, TGFβ KO) have been largely used to achieve long-lasting microglial depletion. However, the serious complications arising from the removal of microglia during early development and the reduced rate of survival have substantially limited the use of such models. Inducible genetic microglial-specific CRE lines have been introduced as a method to overcome these developmental defects and achieve depletion at later developmental stages. However, the specificity of the employed promoters remains questionable. CX3CR1 in the brain is also expressed by perivascular, meningeal, and choroid plexus macrophages (Goldmann et al., 2016), whereas LysM and CD11b have even broader expression among other cell types of the myeloid lineage (Clausen et al., 1999; Ferron and Vacher, 2005). Although microglia represent the major population in the brain expressing these markers, the contribution of other cell types has to be considered.

### Effects of Microglia Depletion on Synapses

All of these depletion models lead to considerable changes in synaptic function *in vivo*. The extent and the duration of such effects, as well as the neural circuits affected, are dependent upon the developmental stages in which the depletion occurs. In fact, different outcomes have been described following embryonic and post-natal depletion.

#### Embryonic Depletion

Depletion of embryonic microglia via CSF1R antibody treatment, and in the PU.1 knockout mouse model, resulted in the defective development of specific and important axonal tracts, as suggested by the abnormal outgrowth of tyrosine hydroxylase (TH)-positive neurons dopaminergic axons in the forebrain, and by the axonal defasciculation in the dorsal corpus callosum (Pont-Lezica et al., 2014; Squarzoni et al., 2014). Early embryonic depletion (E6.5–E7.5) also led to the altered positioning of Lhx6-expressing interneurons in the cortical plate, thus indicating a critical impact on forebrain connectivity (Squarzoni et al., 2014).

#### Post-natal Depletion

Early post-natal depletion of microglia through injection of clodronate liposomes in the rat brain at post-natal day (PND) 2 and PND4 resulted in long-lasting impairments in anxiety, social and locomotor behavior (Nelson and Lenz, 2017). Short and transient microglia ablation in the Cd11b-DTR mouse line at PND3 is sufficient to induce significant neuronal cell death in cortical layer V (Ueno et al., 2013). Embryonic and early post-natal depletion of microglia have a profound effect on CNS architecture, significantly impacting on neuronal viability, neuronal migration, and axonal sprouting. Conversely, microglial depletion at later post-natal stages results in milder developmental effects, mostly involving the fine-tuning of synapses. Microglia depletion at either PND19 or PND30, by using inducible genetic models such as the Cx3cr1^creER/+^; Rosa26^iDTR/+^ revealed a critical function of microglia in regulating baseline remodeling of post-synaptic dendritic spines in the motor cortex (Parkhurst et al., 2013). A significant decrease in both synapse elimination and formation was reported, associated with altered synaptic protein levels and impaired glutamatergic function (Parkhurst et al., 2013). Interestingly, depletion of microglia in adulthood in the same model induced reduction only of synapse formation, but not elimination, in motor learning induced-remodeling. This finding suggests that microglial-mediated pruning of synapses is confined to the first post-natal weeks, whereas the microglial role in supporting synapse formation is maintained across the lifespan (Parkhurst et al., 2013). Consistent with these data, selective microglia depletion in the hippocampus of adult mice by clodronate injections led to impaired spatial learning and social behavior defects. Interestingly, microglia depletion and behavioral effects were both reversible, consistent with a dynamic microglial role (Torres et al., 2016).

*Ex vivo* approaches have allowed further investigations into the role of microglia in refining synapses. Organotypic hippocampal slices depleted of microglia via clodronate liposomes treatment exhibited increased frequency of excitatory post-synaptic currents, consistent with higher synaptic density (Ji et al., 2013a). Replenishing the slices with microglia was sufficient to reverse the depletion effects, thus supporting a critical role for microglia in regulating the number of functional synapses (Ji et al., 2013a).

In summary, depletion models in pre- and post-natal periods demonstrate the essential role for microglia in the maintenance of synaptic function and provide evidence that microglia are critical for proper brain development.

## Microglial Perturbations During Brain Development

The research into synaptic development using depletion models has prompted further investigations to elucidate the physiological function of microglia in refining neural circuits in development. Fundamental insights have come from studies in which the physiological microglial activity was perturbed either by environmental or genetic approaches.

Environmental stimuli such as stress (Merlot et al., 2008), infections (Boksa, 2010), diesel exhaust particles (Bolton et al., 2012, 2014) dietary intake (Giugliano et al., 2006) and un-physiological levels of glucocorticoids (Caetano et al., 2016) are well-characterized inducers of acute and chronic inflammatory responses. Importantly, all of these agents are known to increase the vulnerability to mental diseases characterized by profound synaptic defects (Pedersen and Mortensen, 2001; Volk et al., 2013; Drozdowicz and Bostwick, 2014). Human epidemiological data have shown clear associations between exposure to early-life adverse events and risk of neuropsychiatric conditions later in life (MacMillan et al., 2001), including depression (Agid et al., 1999; St Clair et al., 2015), autism spectrum disorders (ASDs) (Kinney et al., 2008) and psychosis (Varese et al., 2012). Similarly, prenatal infections occurring during the 1st and 2nd trimester of human pregnancy result in higher risk of schizophrenia (Brown et al., 2004) and ASD (Atladottir et al., 2010; Di Marco et al., 2016). These reports suggested a link between immune activation and synaptic development, however, the cellular mechanisms remained elusive.

Further investigations have attempted to dissect the differential contribution of systemic vs. brain immune response. Indeed, recent neuropathological studies in human post-mortem brains have revealed strong microglial activation in neurodevelopmental disorders, such as schizophrenia (de Baumont et al., 2015; Sekar et al., 2016), and ASD (Voineagu et al., 2011; Gupta et al., 2014; Takano, 2015).

While the above studies provided only correlative evidence, data from genetically engineered animal models have demonstrated a causative role for microglia in the pathogenesis of neurodevelopmental disorders. Genetic manipulation affecting specific microglial function resulted in behavioral alterations reminiscent of obsessive-compulsive disorder (Chen et al., 2010; Zhan et al., 2014) and Rett Syndrome (Maezawa and Jin, 2010; Derecki et al., 2012), and in impaired functional brain connectivity reminiscent of autism and other neurodevelopmental disorders (Zhan et al., 2014).

Therefore, the collective evidence from human studies and microglial perturbation animal models (using both environmental and genetic approaches) indicate that altering microglial function during development contributes to the pathogenesis of neurodevelopmental disorders. Given the role of microglia in refining synaptic connectivity, an impaired cross-talk between microglia and synapses might represent a crucial mechanism linking microglial perturbation in early life to mental diseases susceptibility. Below, we review the current literature showing that environmental and genetic perturbations of microglial function during development have acute and long-lasting effects on synapses and behavior.

### Environmental Factors Modulating Microglial Activity

Environmental factors, such as infections, stress and dietary intake are associated with synaptic dysfunction and are linked to increased risk for neurodevelopmental/psychiatric disorders (Marques et al., 2013, 2015; Knuesel et al., 2014; Ajdacic-Gross et al., 2016; Estes and McAllister, 2016). Importantly, all of the above factors have been shown to activate the immune system during early brain development. Recent studies also showed that microglia–synapse contacts are strongly modulated by neural activity, highlighting sensory deprivation as a further regulator of microglial activity. Burgeoning evidence suggests that microglia substantially contribute to the environmental-mediated synaptic defects.

#### Infections

Both viral and bacterial infections have been shown to alter behavior and cognition in experimental animals when administered during critical developmental periods (Boksa, 2010; Meyer, 2014), suggesting profound synaptic remodeling. Importantly, the initial systemic immune response elicited by the infection is followed by a microglial-mediated inflammatory reaction in the brain (Turrin et al., 2001; Puntener et al., 2012). Therefore, there is a growing interest in the role of microglia in infection-mediated synaptic remodeling during development.

##### Viral infection

In a seminal paper, maternal infection of experimental rodents with the influenza virus resulted in deficits in a variety of behavioral displays, including open-field, prepulse inhibition, object recognition and social behavior in the adult offspring (Shi et al., 2003). This study was the first to establish a causal relationship between prenatal viral infection and psychiatric and neurological symptoms in adulthood, which had been previously suggested by a plethora of epidemiological studies (Brown, 2012).

The most widely used model in experimental rodents is the prenatal injection of viral mimetic polyriboinosinic-polyribocytidilic acid (Poly I:C). Poly I:C, a synthetic analog of double-stranded RNA that is recognized as foreign by the rodent immune system, efficiently stimulates an immune response via TLR3 activation. In the mouse species, prenatal immunological stimulation with Poly I:C in early/middle gestation leads to a spectrum of behavioral abnormalities in adulthood, strongly depending on the timing of the infection. Mid-gestational (E9) Poly I:C exposure leads to impairments in pre-pulse inhibition of the acoustic startle reflex and in latent inhibition (Meyer et al., 2005), considered relevant to schizophrenia and ASD. On the other hand, impairments in cognition are elicited following Poly I:C exposure at late gestation (E17) (Meyer et al., 2006). At the molecular level, changes in both pre-and post-synaptic elements, with altered NMDA receptor composition (Forrest et al., 2012), reduced PSD95 and SynGAP density (Giovanoli et al., 2016b), as well as reduced synpatophysin elements (Oh-Nishi et al., 2010; Giovanoli et al., 2016b) have been described in the adult offspring of Poly I:C infected dams. In addition, prenatal Poly I:C injection has been linked to the onset of white matter abnormalities in corticostriatal areas, including myelin-related transcriptional and epigenetic changes, and alterations of myelin water fraction in adulthood (Richetto et al., 2016).

Both acute and chronic immune responses are observed following prenatal Poly I:C injection. Acutely, Poly I:C causes a transient increase in inflammatory mediators in maternal blood and fetal brain (Gilmore et al., 2005; Forrest et al., 2012; Smolders et al., 2015). The cellular origin of these inflammatory mediators is still a matter of debate. Indeed, Pratt et al. (2013) have reported an acute increase in cytokine expression by fetal microglia, suggesting that microglia might be the source of inflammatory cytokines in the brain of prenatally Poly I:C injected mice. However, other authors did not observe acute changes in microglial density or expression of inflammatory markers following prenatal exposure to Poly I:C, in spite of systemic inflammation in the dams (Smolders et al., 2015).

While the inflammatory response in the fetal brain is well-established, its persistence in the post-natal brain is highly controversial. In some studies inflammation was shown to almost completely subside in adulthood, with normal cytokine plasma levels, unaltered microglial density and moderate increase in IL-1β levels in the hippocampus of adult offspring of infected dams at E9 (Giovanoli et al., 2016b) and E14 (Mattei et al., 2014). In others, Poly I:C injections led to sustained cytokine level changes that lasted into adulthood. A comprehensive study by Garay et al. (2013) reported long-lasting, region-specific inflammatory changes in offspring of Poly I:C injected dams (E12.5), across five post-natal ages. In cortical regions, most cytokines were altered at all of the ages analyzed, following a pattern of elevation at birth, decreasing during the early post-natal weeks (PND7-PND30), and elevation again in adulthood (P60) (Garay et al., 2013). Interestingly, cytokine levels in the hippocampus showed a distinct pattern, with mixed direction-switches across ages (Garay et al., 2013). All of the changes in the brain occurred independently of serum cytokine alterations, in the absence of immune cell infiltration, and with no increase in microglia density (Garay et al., 2013).

Importantly, the Poly I:C-induced inflammatory response during development is sufficient to induce long-lasting changes in microglial expression profile and function, but not in microglial numbers. Indeed, recent global transcriptional data have revealed that Poly I:C injection at E14.5 interferes with development of early microglia, shifting them into a more advanced developmental stage (Matcovitch-Natan et al., 2016).

Such early interference has long-lasting effects on microglial function, producing ‘primed’ microglia, with increased susceptibility to activation. In fact, a ‘second hit’ (i.e., sub chronic unpredictable stress) can elicit an exacerbated hippocampal microglial response (with increased soma size, CD68 and IL-1β expression) in the offspring of Poly I:C injected mothers as compared to controls (Giovanoli et al., 2016a).

The molecular mediators of Poly I:C effects on both the structure and the function of the developing CNS are not fully elucidated; however, inflammatory cytokines, including IL-6 and IL-1β, are likely to be crucial players. IL-6 release has been shown to mediate the behavioral effects of viral prenatal infections. Injection of IL-6 into pregnant dams at E12 induced pre-pulse inhibition and latent inhibition deficits in adulthood, similar to Poly I:C treatment. Importantly, genetic or pharmacological blockade of IL-6 fully prevented Poly I:C-induced gene expression changes and behavioral alterations (Smith et al., 2007). In addition, recent evidence suggests that IL-1β might be involved in the effects of prenatal Poly I:C on white matter in adulthood. Intraperitoneal injections of IL-1β in newborn mice (PND1-PND4) is sufficient to largely disrupt the developmental program of white matter (Favrais et al., 2011), in line with the observations of Richetto et al. (2016) in Poly I:C-challenged offspring. Recent studies found that the anti-inflammatory drug minocycline prevented the effect of prenatal Poly I:C administration on behavior in adulthood (Mattei et al., 2014; Zhu et al., 2014; Giovanoli et al., 2016a), further indicating a causative relationship between inflammation and synaptic dysfunction.

##### Bacterial infections

Systemic injections into pregnant dams or pups of the gram-negative bacterial component lipopolysaccharide (LPS), or inactivated *Escherichia coli*, are commonly used models to study central effects of pre- and early post-natal bacterial infections.

Maternal immune activation with LPS resulted in increased anxiety (Hava et al., 2006) and decreased social behavior in the male offspring (Kirsten et al., 2010), phenotypes relevant to ASD. The behavioral alterations were accompanied by glutamatergic remodeling in the hippocampus, with increased AMPAR contribution to excitatory neurotransmission (Roumier et al., 2008) and increased spine density (Fernandez de Cossio et al., 2016).

A prenatal LPS injection caused acute and long-lasting effects on microglia. LPS administration at E15–16 resulted in immediate up-regulation of iNOS expression in microglia in the embryos (Cunningham et al., 2013), indicating acute activation. Such alterations persist during early post-natal periods, with increased microglial density in hippocampus (Roumier et al., 2008) and substantia nigra (Ling et al., 2006) of prenatally LPS-injected mice analyzed at PND0. Importantly, up-regulation of OX-6^+^ microglial cells in the substantia nigra was shown to be sustained until PND84, and to be further exacerbated by a second hit of LPS injection in adulthood, suggesting enduring effects on prenatal infection on microglia (Ling et al., 2006).

Several reports support the notion that prenatal bacterial infection interferes with specific physiological functions of microglia during development. Interestingly, synaptic remodeling induced by LPS prenatal injection is phenocopied in models of microglial depletion, pointing to a loss of homeostatic function.

Roumier et al. (2004, 2008) have shown that mice carrying a loss of function mutation in the DAP12 gene (a signaling protein transiently expressed by microglia at birth) recapitulate the glutamatergic remodeling seen with LPS injection. More recently, Squarzoni et al. (2014) have shown that maternal immune activation with LPS induces alterations in the laminar positioning of LHX-6^+^ interneurons similar to what was observed via depletion of microglia. The exact mechanisms by which prenatal bacterial infection interferes with synapse–microglial interaction are still unclear, but recent evidence suggests a role for CX3CR1 signaling. In fact, prenatally LPS-infected rats display reduced hippocampal CX3CR1 expression along with increased spines in the dentate gyrus (Fernandez de Cossio et al., 2016). It could be speculated that the LPS induced-downregulation of the CX3CR1 receptor might alter the physiological pruning of synapses. However, further work is required to test this hypothesis.

Early post-natal infection with LPS has been shown to induce anxiety-like behaviors (Sominsky et al., 2012) and impaired cognitive function in adult rats (Pang et al., 2016). LPS injection in rat pups at PND5 results in acute microglial activation with increased numbers of Iba1^+^ cells in the hippocampus and up-regulation of both M1 and M2 markers (Smith et al., 2014; Pang et al., 2016). Iba1^+^ cell numbers normalized by PND21, but M1 marker expression was still subtly altered (Smith et al., 2014). Furthermore, increased Iba1 immunoreactivity was observed in the dentate gyrus of PND85 neonatally infected rats (Sominsky et al., 2012). These results suggest that behavioral deficits might be due to both acute and long-lasting effects of neonatal LPS injection on microglia.

*Escherichia coli* administration in PND4 rats induced mild behavioral perturbations in adulthood, including hippocampal-dependent deficits in the reversal phase of the Morris Water Maze task (Williamson and Bilbo, 2014) and impaired motor coordination (Lieblein-Boff et al., 2013). Effects on inflammatory markers are short and transient in this model, with up-regulation of IL-1β and CD11b expression in the hippocampus that normalizes by 72 h post-injection (Bilbo et al., 2005). However, the immune system of post-natally *E. coli*-infected mice has been shown to be primed, and displays an exacerbated response to a ‘second hit’ with a low-dose of LPS, in adulthood (Bilbo et al., 2005; Williamson et al., 2011). Importantly, the immune alterations caused by the ‘second hit’ with LPS were accompanied by severe memory impairments, indicating a crucial role for primed microglia in mediating cognitive deficits. Importantly, blocking microglial activation with minocycline or caspase1 inhibitor immediately before a ‘second hit’ of LPS in adulthood prevented the appearance of cognitive deficits in neonatally infected rats (Bilbo et al., 2005; Williamson et al., 2011). It is crucial to note that *E. coli* injections at later post-natal time points, such as PND30, do not result in exacerbated microglial response or altered behavioral display following a ‘second hit’ with LPS in adulthood (Bilbo et al., 2006). These findings further support the notion that the acquired vulnerability to the second hit is not a general sensitizing event, but it is strictly dependent on the developmental stage in which the first infection occurs (Bilbo and Schwarz, 2009, 2012).

#### Stress

Intermittent and unpredictable maternal separation, as well as stressful manipulations of pregnant dams (via restraint, sleep deprivation, or light exposure), are common experimental models of early-life adverse events and developmental stress. In striking similarity with human data, the early-stressed pups go on to develop syndromes later in life that resemble depression (Mintz et al., 2005; Ruedi-Bettschen et al., 2005), and anxiety (Vallee et al., 1997); for a review (Nishi et al., 2014). In recent years an increased interest has arisen on the role of microglia in early-stress-induced remodeling of cortical and hippocampal synapses. Indeed, different models of perinatal stress have been shown to induce both acute and long-lasting effects on microglia activity.

Prenatally stressed mice displayed higher numbers of ramified microglia in several brain regions analyzed at PND1, including parietal, entorhinal and frontal cortices, septum, basal ganglia, thalamus, medulla oblongata, and internal capsula (Gomez-Gonzalez and Escobar, 2010). Primary cultures of microglia isolated from prenatally stressed rats showed increased release of pro-inflammatory cytokines, including IL-1β, IL-18, TNF-α and IL-6, and reduction of IGF-1 (Slusarczyk et al., 2015). Such cytokines, and in particular IL-18 and TNF-α, were shown to directly increase neuronal excitability via up-regulation of voltage-activated Na^+^ currents (Igelhorst et al., 2015; Klapal et al., 2016). The microglial effects have been shown to be mostly transient, with microglia morphology returning to normal by PND10 (Gomez-Gonzalez and Escobar, 2010). However, prenatally stressed mice, when examined in adulthood, showed hippocampal microglial alterations, with a higher density of Iba1^+^ cells, enlargement of soma, increased pro-inflammatory expression and heightened response to a second hit with LPS injection (Diz-Chaves et al., 2012, 2013). Similar findings were reported in rats, with adult prenatally stressed subjects displaying increased expression of microglial activation markers, and increased Iba1^+^ microglial density in the hippocampus and frontal cortex (Slusarczyk et al., 2015; Zhao et al., 2015). These results suggest that prenatal stress can induce long-lasting effects on microglial function in the hippocampus and cortex.

More subtle effects have been observed in models of early post-natal stress. Maternal deprivation during the first 2 weeks of life was associated with acute effects on hippocampal microglia, including larger soma size, increased release of IL-1β (Roque et al., 2016) and increased microglial surface area (Delpech et al., 2016). Delpech et al. (2016), however, reported that alterations in microglial morphology were transient and parameters normalized later at PND28. Analysis of PND28 hippocampi from mice that experienced early stress revealed that, in spite of apparently normal morphology, microglia had altered transcriptional activity of PU1, Creb and Sp1, resulting in gene expression changes and increased phagocytic activity (Delpech et al., 2016). Long-lasting effects of early-life stress were also reported in other brain areas. Increased microglia motility was observed in the sensorimotor cortex of adult mice that had experienced maternal deprivation (Takatsuru et al., 2015). In this study, the authors observed a positive correlation between microglial motility and nociceptive threshold, suggesting a functional link between altered microglia activity during development and synaptic plasticity in adulthood.

Minocycline treatment can efficiently prevent behavioral deficits that follow prenatal stress, normalizing, in parallel, microglial expression of pro- and anti-inflammatory markers in the hippocampus (Zhao et al., 2015). These findings suggest that long-lasting microglial alterations might mediate the behavioral consequences of early stress.

The link between microglia and stress has been convincingly shown in adult mice. Anti-inflammatory treatment ameliorated chronic stress-induced behaviors (Hinwood et al., 2012, 2013; Kreisel et al., 2014; Fuertig et al., 2016). Furthermore, mice lacking the microglial-specific CX3CR1 receptor showed resilience to stress-induced depressive behavior (Hellwig et al., 2016; Milior et al., 2016). Therefore, microglia can play a crucial role in mediating vulnerability to stress and its downstream effects on synaptic circuits. However, the molecular mechanisms by which microglia mediate synaptic and behavioral changes in early-stress models are far from been understood.

#### Dietary Intake

Imbalance in dietary intake has been convincingly associated with cognitive function. High-fat diet (HFD) consumption is associated with cognitive impairment, increased aggression and altered social- and reward-related behaviors in mice (Hilakivi-Clarke et al., 1996; Pistell et al., 2010; Takase et al., 2016). A mouse model of n-3 polyunsaturated fatty acids (PUFAs) deficiency resulted in defective spatial learning and memory tasks, with increased depressive-like behavior (Larrieu et al., 2012; Moranis et al., 2012).

Both the imbalance in PUFAs content and the long-term exposure to HFD lead to dysregulation of inflammatory processes (Ortega et al., 1997; Pistell et al., 2010; Sharma et al., 2012; Valdearcos et al., 2014; Baufeld et al., 2016; Guillemot-Legris et al., 2016; Gzielo et al., 2016). Interestingly, docosahexaenoic acid (DHA), one of the most abundant PUFAs in the brain and a potent immunomodulator, was shown to prevent LPS-induced cytokine production in microglia, by directly inhibiting the surface presentation of CD14 and TLR4 (De Smedt-Peyrusse et al., 2008). A growing literature provides evidence for a direct role of DHA in regulating microglial activity (Antonietta Ajmone-Cat et al., 2012; Chen et al., 2014; Chang et al., 2015; Baufeld et al., 2016; Hao et al., 2016; Hopperton et al., 2016; Tremblay et al., 2016).

The effects of dietary imbalance during development have been recently investigated. Dietary intake was shown to act as a modulator of microglial behavior in the perinatal brain in studies where female mice were fed throughout gestation and lactation with n-3 PUFAs deficient or a control diet. Mice raised under n-3 PUFAs deficiency exhibited altered levels of microglial phenotypic markers and defective microglia motility at PND21. This defective microglial activity was associated with increased pro-inflammatory cytokine expression, and altered expression of plasticity-related genes in neurons (Madore et al., 2014). Overall these findings indicate that dietary intake, specifically in relation to PUFA content, might drastically impact on microglial properties. The consequent microglial-mediated increase in inflammation, when occurring early in development, may contribute to neurodevelopmental disorders (Bolton and Bilbo, 2014).

The mechanisms linking dietary intake to microglial activation are beginning to be elucidated. Erny et al. (2015) have recently shown that gut microbiota control maturation and function of microglia, possibly via release of short-chain fatty acids (SCFAs: microbiota-derived bacterial fermentation products). Germ-free mice and antibiotic treated mice displayed altered microglia expression and morphology consistent with immature phenotype; the results were phenocopied in mice deficient for the SCFA receptor FFAR2 (Erny et al., 2015). Hence, evidence exist that dietary intake can modulate microglial activity, and at least some of this interaction might be mediated via host microbiota (Smith, 2015; Campos et al., 2016; Erny et al., 2017).

#### Neural Activity-Dependent Modulation of Microglia

Increasing neural activity by pharmacological blocking of inhibition, by AMPAR or by NMDAR activation (Fontainhas et al., 2011; Dissing-Olesen et al., 2014), but not by electrical stimulation (Wu and Zhuo, 2008), results in enhanced microglial motility and extension of microglial processes. Conversely, GABAergic activation leads to a reduction in microglial motility (Fontainhas et al., 2011). Many studies support the theory that microglia–synapse contacts and engulfment of synaptic material are strongly modulated by neural activity. Apposition between microglia and synaptic terminals increase following neuronal activity, and correlate with higher rate of synaptic elimination upon ischemia (Wake et al., 2009) and in an experience-dependent manner (Tremblay et al., 2010). Similarly, microglia-mediated engulfment of synaptic inputs in the early developing dorsal lateral geniculate nucleus is regulated by neural activity: increasing upon inhibition with tetrodotoxin or stabilizing with forskolin activation (Schafer et al., 2012).

Indeed, environmental manipulations that either increase or decrease neuronal activity have been shown to impact on the microglial phenotype and its interaction with dendritic spines. Environmental enrichment (EE) is a well-established experimental paradigm of neuronal stimulation, resulting in a brain derived neurotrophic factor (BDNF)-dependent increase in the number of spines, physical rewiring of the brain and improvement in several cognitive domains (van Praag et al., 2000). Exposure to an enriched environment has also been shown to promote microglial branching (Xu et al., 2016) and profoundly affect neuroimmune functions in the hippocampus (Williamson et al., 2012). Importantly, LPS injection abrogated the EE-induced increase in BDNF production, suggesting that microglia are not simply bystanders, but are actively involved in the beneficial effects of EE (Williamson et al., 2012). On the other hand, deprivation of a specific sensory input (e.g., vision) during critical developmental periods also results in dramatic neuronal remodeling at both the synaptic and circuit levels. Microglia have been shown not only to respond to sensory deprivation, but also to mediate some of its neuronal effects. Following dark adaptation (a paradigm resulting in increased spine motility and turnover in primary visual cortex, Majewska and Sur, 2003), Tremblay et al. (2010) found subtle changes in the behavior of microglia in the primary visual cortex of juvenile mice. The observed changes included an expansion of microglial processes, an increased occurrence of cellular inclusions (suggesting phagocytosis), an increased frequency of contact with synaptic clefts, and an increased apposition with synaptic elements, with overall reduced motility (Tremblay et al., 2010). These results are largely confirmed by Sipe et al. (2016) in a model of monocular deprivation (MD), where sensory inputs are abrogated from one eye only. In this model, the authors observed an increase in the contacts between microglia and spines, occurring at day 1 post MD. This increased interaction was followed by active engulfment of synaptic material by microglia 4 days after MD. These data suggest that microglia are capable of sensing the altered neuronal activity and can respond by remodeling synapses (Sipe et al., 2016). Importantly, in MD models, neuron–microglia interaction was shown to significantly contribute to the functional plasticity that follows sensory deprivation. In fact, MD normally results in the full shift of ocular dominance from the closed eye to the open eye within 2 days (Wiesel and Hubel, 1963). However, this effect was abrogated in mice either lacking the P2Y12 receptor, or receiving a specific P2Y12 inhibitor (Sipe et al., 2016). Since this receptor is exclusively expressed in CNS microglia, crucially mediating the neuron–microglia cross-talk (Sasaki et al., 2003; Haynes et al., 2006), the above results indicate that sensory deprivation requires microglia to rewire the visual cortex. How P2Y12 modulates ocular dominance, and whether this is mediated via spine remodeling, remains to be elucidated.

A reciprocal regulation between microglia and neural activity has also been shown in the zebrafish optic tectum, where microglia contacts with active neurons are more frequent, and result in neuronal silencing in a contact-dependent mechanism (Li et al., 2012). These findings indicate that neural activity is shaping brain connectivity also through the critical contribution of microglia.

### Genetic Factors Modulating Microglial Activity

The use of genetically modified mouse models and the recent advent of Cre lines selective for microglia have been instrumental in dissecting the role of specific genes in modulating microglial activity. Altering the function of genes involved in the regulation of key executive microglial tasks, such as motility, migration, phagocytosis and cytokine release, affects a broad spectrum of microglial-mediated cellular processes, which occur in the developing brain. Several such alterations are linked to the onset of behavioral symptoms reminiscent of neurodevelopmental and psychiatric disorders in humans, indicating a key role for microglia in such diseases.

**DAP12** is a transmembrane protein associated with the TREM2 receptor, with specific microglial expression, and is up-regulated in the developing brain (Aoki et al., 2000; Roumier et al., 2008). Mouse models expressing a mutated form of DAP12 lacking the expression of the functional protein were one of the first examples of selective microglia manipulation significantly impacting on synaptic function. DAP12 KO mice displayed defective mIPSC (miniature inhibitory post-synaptic currents), impaired AMPAR accumulation at the post-synaptic site, and showed a drastic reduction in BDNF-specific TrkB receptor. Such impairments translated into defective synaptic transmission, thus likely to affect brain wiring (Kaifu et al., 2003; Roumier et al., 2004). In addition, the positioning of Lh6-expressing interneurons in the cortical plate of E18.5 embryos and P7 mice was impaired in the DAP12 KO models, suggesting that microglia may support the functioning of neural circuits by contributing to the maintenance of inhibitory/excitatory balance (Squarzoni et al., 2014). Adult DAP12 KO mice displayed phenotypes relevant to schizophrenia and ASD, such as reduced startle reflex in response to acoustic stimuli and reduced prepulse inhibition, suggesting an impairment of sensorimotor gating (Kaifu et al., 2003).

**CX3CR1** is a G_i_-protein coupled receptor, encoded by the *Cx3cr1* gene, expressed mostly by microglia in the brain (Jung et al., 2000; Mizutani et al., 2012). Its ligand CX3CL1, also known as fractalkine, is on the other hand largely expressed in neurons (Tarozzo et al., 2003). The fractalkine signaling has been extensively investigated in recent years, with accumulating evidence supporting its role in controlling fundamental microglia functions (Limatola and Ransohoff, 2014; Paolicelli et al., 2014; Arnoux and Audinat, 2015). The loss of the fractalkine signaling significantly affects basic microglial properties, such as voltage-dependent K^+^ currents, processes ramification and ATP-induced migration (Pagani et al., 2015). The brains of Cx3cr1 KO embryos displayed extensions of TH-positive dopaminergic neurons, selectively in the subpallium, but not in the midbrain, and showed defective positioning of Lh6-expressing interneurons in the neocortical plate (Squarzoni et al., 2014). Cx3cr1 KO brains at early post-natal stages also showed a significant increase in cell death in the cortical layer V, phenocopying the effects of microglia depletion via DTR expression (Ueno et al., 2013). Hoshiko et al. (2012) showed that the lack of the Cx3cr1 was associated with delayed maturation of the developing synapses in the barrel cortex, specifically affecting the NMDAR composition. These findings confirmed previous studies reporting causal effects on synaptic plasticity with modulation of Cx3Cl1/Cx3cr1 signaling (Ragozzino et al., 2006; Maggi et al., 2011; Rogers et al., 2011). Cx3cr1 KO mice displayed a transient reduction in microglial numbers in the developing hippocampus, and showed defective synaptic pruning, resulting in synaptic deficits and long-lasting impairments in social behavior and brain connectivity (Paolicelli et al., 2011; Zhan et al., 2014). The lack of fractalkine signaling was associated with reduced levels of free Insulin-like Growth Factor 1 (IGF1) in the mouse cortex at PND5, ultimately affecting the survival of corticospinal and callosal neurons (Ueno et al., 2013). Overall, these observations indicate that the defective microglial function induced by the lack of Cx3cl1/Cx3cr1 signaling significantly affects brain development, with long-lasting consequences on neural circuits and brain function.

**BDNF** Microglial contribution to synaptic plasticity and function has been elegantly demonstrated through an inducible conditional knockout (cKO) mouse line, where BDNF was selectively depleted from microglia (Parkhurst et al., 2013). BDNF influences a large variety of cellular processes, ranging from survival, to apoptosis, cellular morphology, and synaptic plasticity (Hohn et al., 1990; Lu et al., 2005; Park and Poo, 2013). Along with neurons, microglia also represent a source of neurotrophin in the brain (Elkabes et al., 1996; Elkabes et al., 1998; Coull et al., 2005; Trang et al., 2011). Mice lacking microglial BDNF displayed altered synaptic protein levels and impaired spine formation during motor learning tasks, which resulted in the absence of training-induced improvement in motor behavior performance, suggesting an important role for microglial derived BDNF in modulating synaptic plasticity (Parkhurst et al., 2013).

**The Complement system** is a complex innate immune surveillance system, with important functions in inflammation, pathogen defense and host homeostasis (Zabel and Kirsch, 2013; Merle et al., 2015). Complement molecules such as C1q and C3, are up-regulated in the early post-natal brain, and have been implicated in synaptic remodeling during the development of the retinogeniculate system (Stevens et al., 2007). C1q and C3, closely apposed to synapses, work as ‘eat-me’ signals for the synaptic terminals to be removed. Loss of function approaches, such as genetic knockout mouse lines, resulted in increased synapse number, suggesting that the absence of such molecules could impair synaptic pruning by microglia, leading to enhanced connectivity and epilepsy (C1q KO model, Chu et al., 2010) and decreased synaptic engulfment (C3 KO or Complement Receptor 3, CR3 KO, Schafer et al., 2012). Recently, microglial CR3 activation was found to trigger long term synaptic depression (LTD), upon the combination of hypoxia and inflammatory stimuli, via NADPH oxidase. This finding suggests that microglial CR3-triggered LTD may underlie synaptic dysfunction (Zhang et al., 2014). Further investigations are required to elucidate the possible occurrence of such mechanisms during early brain development.

**Progranulin (PGRN)** is a protein encoded by the progranulin gene (GRN), and it is implicated in the regulation of phagocytosis and the release of pro-inflammatory cytokines in microglia and macrophages (Yin et al., 2010; Kao et al., 2011; Martens et al., 2012). GRN is associated with frontotemporal lobar degeneration, and microglia lacking GRN are shown to have an aberrant expression profile at 18 but not at 4 months of age (Lui et al., 2016). However, the same study showed that primary microglia isolated from neonatal GRN KO mice expressed more abundant levels of C1q and C3 complement molecules, described to mediate synaptic pruning (see above). *In vitro* systems assessing the functional implication of GRN KO in neonatal microglia, based on primary neuronal–microglia co-culture, revealed a significant increase in synaptic pruning, and provided evidence for C1qa-tagged synaptic puncta within microglial phagocytic structures (Lui et al., 2016). These findings indicate that an aberrant complement expression by microglia, combined with an enhanced phagocytosis, is detrimental for the synaptic function.

**ATG7** (autophagy related gene 7) is an E1-like activating enzyme required for cytoplasm – vacuole transport, and is essential for autophagy. Kim et al. (2016) recently showed that microglial autophagy is involved in synaptic refinement. Microglia lacking Atg7 displayed impaired degradation of synaptosomes *in vitro*. Deletion of Atg7 from myeloid cell-specific lysozyme M-Cre mice resulted in increased dendritic spines and synaptic markers, and was associated with long-lasting deficits in sociability, with no changes in social recognition. In addition, increased repetitive behaviors were observed in mice lacking microglial Atg7, indicating that deficient microglial function in the developing brain might lead to ASD-like behaviors (Kim et al., 2016).

Overall these findings, based on selective manipulation of microglia, indicate that genes regulating microglial functions can have a critical impact on synaptic refinement and might be involved in neurodevelopmental disorders.

## Conclusions

There is substantial evidence that microglia are important cellular players during early brain maturation. Microglial activity can be modulated by a variety of environmental and genetic factors. Alterations of microglial function induced during development by agents such as stress and infections have been extensively associated with defective synaptic maturation and impairment in brain connectivity. The release of pro-inflammatory, neurotoxic cytokines in response to these factors has been for long time considered the most important microglial-mediated mechanism leading to synaptic defects. In addition to this gain-of-toxicity, the recent literature reviewed here indicates that early microglia perturbation also induces a loss-of-function effect. In fact, many of the synaptic defects reported upon microglial depletion could be phenocopied in mouse models where the physiological activity of microglia is heavily altered. Pont-Lezica et al. (2014) showed that three different models of altered microglial activity (genetic depletion in PU.1KO mice, loss-of-function in the DAP12KO mouse line, and maternal inflammation by prenatal LPS injection) all resulted in a comparable defect of the corpus callosum development.

Consistently, Squarzoni et al. (2014) found that perturbing microglial activity using multiple mouse models, including cell-depletion approaches and Cx3cr1KO, CR3KO, and DAP12KO mice led to similar defects in the outgrowth of dopaminergic axons in the forebrain and in the laminar positioning of subsets of neocortical interneurons.

These findings indicate that perturbations of microglial activity during development interfere with physiological cross-talk between microglia and synapses. One could speculate that impaired synaptic pruning and reduced release of trophic factors might be the major mediators of the synaptic defects. However, the exact molecular mechanisms remain elusive and require further investigations.

The specific timing of microglial perturbation during development is a critical factor in dictating the outcome and duration of the effects on synaptic functions. Indeed, the precise time of manipulation might affect microglial as well as neuronal developmental programs.

Microglial and macrophagic development follows a stepwise program orchestrated by the activity of specific transcription factors, which dictate discrete transcriptional phases (Hagemeyer et al., 2016; Matcovitch-Natan et al., 2016). Recent data from mouse studies suggest that this partitioning consists primarily into three major stages: early microglia until E14, pre-microglia from E14 to a few weeks after birth, and adult microglia from a few weeks after birth onward (Matcovitch-Natan et al., 2016). Coordinated transcriptional events control the transitions through such microglial developmental stages and are probably due to changes in the microenvironment of the CNS. Timed perturbations of this tight shift regulation lead to altered expression patterns, thus affecting key microglial functions.

Neuronal maturation is also strictly dependent on the developmental stage. Neural circuits are refined by experience during specific time windows, defined as critical periods, early in post-natal life (Hensch, 2004). In addition, the timing of neuronal and microglial maturation is sexually dimorphic (Lenz and McCarthy, 2015; Hanamsagar and Bilbo, 2016). It could be speculated that the lack of microglial function might result in different outcome according to the sex and to the developmental stage, in which the manipulation occurs. Further studies are warranted to elucidate the effects of timing and sex on microglial sculpting of synapses.

**FIGURE 1:**
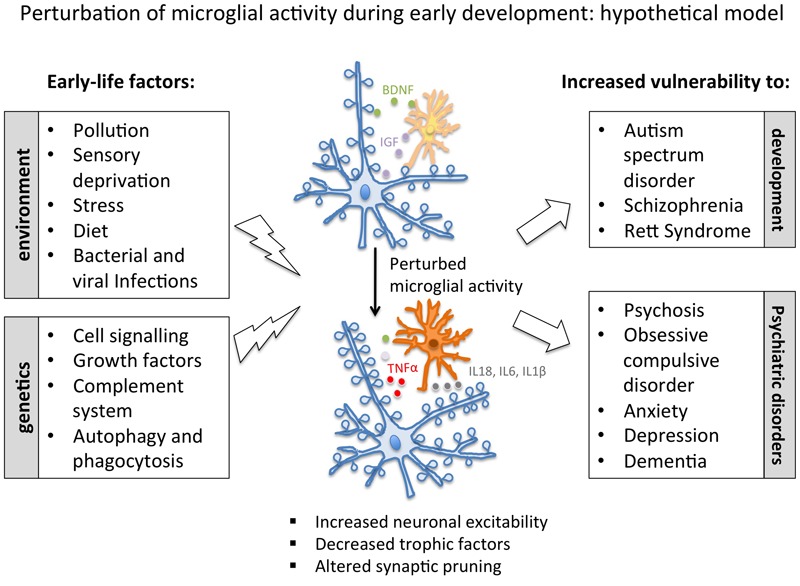
**Schematic representation of the major environmental and genetic factors described to modulate microglial activity.** According to this hypothetical model, microglial-mediated altered circuit formation (as induced by early infections, stress and imbalanced diet, or by genetic causes) could contribute to higher vulnerability to a number of neurodevelopmental and psychiatric disease. BDNF, brain-derived neurotrophic factor; IGF, insulin-like growth factor 1; IL, interleukin; TNFα, tumor necrosis factor α.

In summary, environmental challenges as well as manipulations of microglial genes have been shown to perturb microglial activity, and to affect synapses (see **Tables 2, 3**). Both loss of physiological function and gain-of-toxicity in microglia, when occurring during development, can cause profound alterations in brain wiring. Abnormal synaptic pruning (insufficient or exaggerated) as well as reduced release of trophic factors (such as BDNF and IGF1) can induce aberrant or dysfunctional circuit formation. On the other hand, the release of pro-inflammatory cytokines such as IL-18 and TNFα can directly modulate synaptic activity, by up-regulating voltage-activated Na^+^ currents, thus increasing neuronal excitability. Such alterations occurring in early-life have been shown to have long-lasting effects on brain function, with deficits in behavior and cognition emerging in juvenile age and adulthood (**Figure 1**).

**Table 2:** Environmental factors shown to affect synapses and behavior via microglia.

	Model	Timing	Reported effects in adulthood	Publications
Infections	Viral (Poly I:C)	Prenatal	IL-6 mediated behavioral alterations	Smith et al., 2007
			IL-1β mediated white matter disruption	Favrais et al., 2011
			Minocycline prevents Poly I:C-induced behavioral abnormalities	Mattei et al., 2014; Zhu et al., 2014; Giovanoli et al., 2016a
	Bacterial (LPS)	Prenatal	Reduced hippocampal CX3CR1 expression; increased spines hippocampus	Fernandez de Cossio et al., 2016
	Bacterial (*E. coli*)	Post-natal	Primed microglia with exacerbated response to second hit; minocycline and caspase1 inhibitor prevent the appearance of cognitive deficits in neonatally infected mice subjected to a second hit	Bilbo et al., 2005; Williamson et al., 2011
Stress	Sleep deprivation	Prenatal	Minocycline prevents behavioral deficits	Zhao et al., 2015
	Maternal deprivation	Early post-natal	Increased microglia motility correlates with nociceptive threshold	Takatsuru et al., 2015
Dietary imbalance	n-3 PUFAs deficient diet	Prenatal and early post-natal	Defective microglia motility associated with altered expression of plasticity-related genes in neurons	Madore et al., 2014
Sensory deprivation	Monocular deprivation	Post-natal	Mice lacking P2Y12 receptor do not display full shift of ocular dominance	Sipe et al., 2016

*The table summarizes the main environmental factors that have been shown to affect neuronal circuits and behavior via immune mechanisms and/or microglia. The reader should consult Section “Environmental Factors Modulating Microglial Activity” for details and discussion.*

**Table 3:** Microglial genes shown to affect synapses and behavior.

	Protein manipulated	KO type	Reported effects	Publications
Transmembrane proteins	DAP12	Constitutive	Reduced startle reflex to acoustic stimuli and reduced pre-pulse inhibition; Defective mIPSCs; impaired AMPA receptor accumulation: reduction in TrkB receptors; defective synaptic transmission; defective positioning of Lh6-expressing interneurons in cortical plate of E18.5	Kaifu et al., 2003; Roumier et al., 2004; Squarzoni et al., 2014
	CX3CR1	Constitutive	Defective positioning of Lh6-expressing interneurons in cortical plate of E18.5; increased cell death in cortical layer V; delayed maturation of synapses in barrel cortex; defective synaptic pruning and long-lasting impairments in social behavior and brain connectivity	Ragozzino et al., 2006; Maggi et al., 2011; Paolicelli et al., 2011; Rogers et al., 2011; Ueno et al., 2013; Squarzoni et al., 2014; Zhan et al., 2014
Complement	C1q	Constitutive	Enhanced connectivity and epilepsy	Chu et al., 2010
	C3 or CR3	Constitutive	Decreased synaptic engulfment	Schafer et al., 2012
Trophic factors	BDNF	Inducible conditional KO in CX3CR1-expressing cells	Altered synaptic protein levels; impaired spine formation and elimination; lack of training-induced improvement in motor behavior performance	Parkhurst et al., 2013
Phagocytosis and autophagy-related proteins	Progranulin	Constitutive	Increased synaptic pruning of C1q-tagged synapses	Lui et al., 2016
	ATG7 (autophagy related gene 7)	Conditional in lyzsozyme M-expressing cells	Increased dendritic spines; increased synaptic markers; long-lasting effects in sociability and repetitive behaviors	Kim et al., 2016

*The table summarizes the main genetic factors that have been shown to affect neuronal circuits and behavior via microglia. The reader should consult Section “Genetic Factors Modulating Microglial Activity” for details and discussion.*

Overall, there is substantial evidence that microglial dysfunction in early development leads to defective synaptic maturation and activity. Synaptic defects are a common feature shared by several neurodevelopmental and psychiatric disorders, such as ASD and schizophrenia. Thus, alterations in the physiological activity of microglia might be implicated in the pathogenesis of such diseases. A better understanding of the underlying molecular mechanisms might yield to the identification of novel pharmacological targets for therapeutic interventions.

## Author Contributions

All authors listed, have made substantial, direct and intellectual contribution to the work, and approved it for publication.

## Conflict of Interest Statement

The authors declare that the research was conducted in the absence of any commercial or financial relationships that could be construed as a potential conflict of interest.
